# Solid-Phase Synthesis of Oligodeoxynucleotides Containing *N*^4^-[2-(*t*-butyldisulfanyl)ethyl]-5-methylcytosine Moieties

**DOI:** 10.3390/molecules15085692

**Published:** 2010-08-18

**Authors:** Sónia Pérez-Rentero, Alejandra V. Garibotti, Ramón Eritja

**Affiliations:** Institute for Research in Biomedicine (IRB Barcelona), CIBER-BBN Networking Centre on Bioengineering, Biomaterials and Nanomedicine, Institute for Advanced Chemistry of Catalonia (IQAC) CSIC, Baldiri Reixac 10, E-08028 Barcelona, Spain; E-Mails: sonia.perez@irbbarcelona.org (S.P.-R.); alejandra.garibotti@irbbarcelona.org (A.V.G.); ramon.eritja@cid.csic.es (R.E.)

**Keywords:** solid-phase synthesis, oligonucleotides, DNA, thiol, oligonucleotide conjugates

## Abstract

An efficient route for the synthesis of the phosphoramidite derivative of 5-methylcytosine bearing a *tert*-butylsulfanyl group protected thiol is described. This building block is used for the preparation of oligonucleotides carrying a thiol group at the nucleobase at the internal position of a DNA sequence. The resulting thiolated oligonucleotides are useful intermediates to generate oligonucleotide conjugates carrying molecules of interest at internal positions of a DNA sequence.

## 1. Introduction

Oligonucleotides bearing thiol groups are useful intermediates for different applications, including conjugation of reporter molecules [1,2] and peptides [3,4,5], structural studies [6,7,8] or immobilization on surfaces [9] and preparation of DNA-functionalized gold nanoparticles [10,11]. In most of cases, the thiol groups are introduced at the 5’- and 3’-ends in order to maintain the hybridization properties of the oligonucleotides [1,2], but thiol groups have also been introduced at the phosphate bonds [12], at the 2’-position [13] and at the nucleobases [5,14,15,16]. 

We are interested in the incorporation of thiol groups into two-dimensional DNA lattices [17] by introducing a single thiol group per DNA unit. Thiol groups have a strong affinity for gold surfaces and they can be used for the introduction of peptides, proteins and a large number of molecules functionalized with maleimido- or bromo- or iodo-acetyl groups. Previous work has demonstrated that bidimensional DNA arrays carrying *N*^4^-[2-(t-butyldisulfanyl)ethyl]cytosine residues at the apex of the topological markers can be deposited on gold surfaces [17]. The starting material for the synthesis of the 2’-deoxycytidine phosphoramidite building block carrying the *N*^4^-(*t*-butyldisulfanyl)ethyl group was 2’-deoxyuridine [15], which is expensive and the synthetic route is long and tedious. We become interested in optimizing the preparation of a similar building block using thymidine instead of 2’-deoxyuridine as starting material. Here, we describe a straightforward synthesis of the 2’-deoxy-*N*^4^-[2-(t-butyldisulfanyl)ethyl]-5-methylcytidine phosphoramidite building block starting from thymidine. During the use of the new building block a side product coming from the oxidation of the *N*^4^-(*t*-butyldisulfanyl)ethyl group was detected. Replacement of iodine solution with a *t*-butylhydroperoxide one eliminates this side reaction. Finally, the preparation of several fluorescent DNA conjugates using oligonucleotides containing the N^4^-mercaptoethyl-5-methylcytosine is described. 

## 2. Results and Discussion

**Scheme 1:**
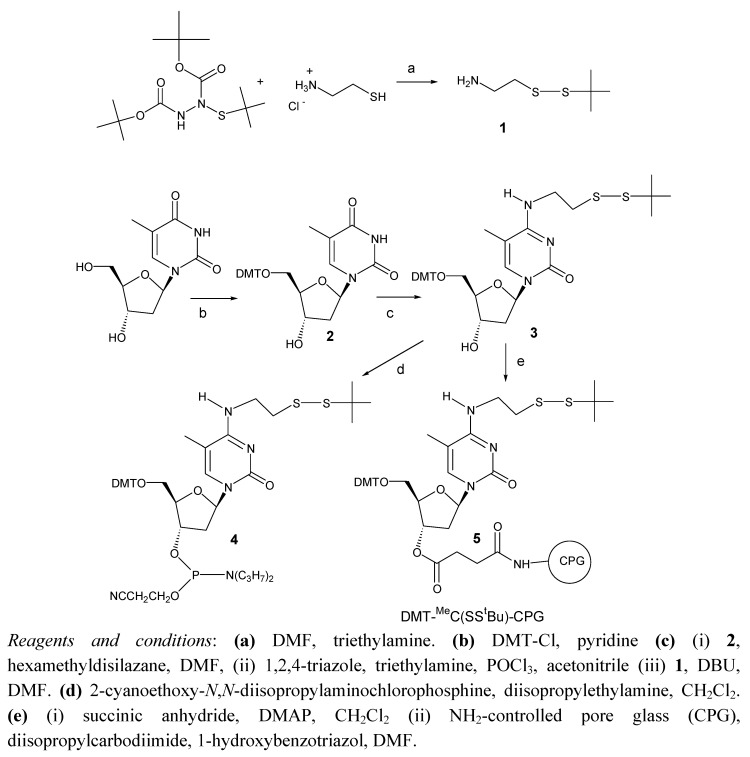
Synthesis of the protected phosphoramidite derivative of 2’-deoxy-[2-(*t*-butyldisulfanyl)ethyl]-5-methylcytidine.

The synthesis of the protected phosphoramidite derivative of 2’-deoxy-[2-(t-butyldisulfanyl)ethyl]-5-methylcytidine is illustrated in Scheme 1. First, 2-aminoethyl-*tert*-butyl disulfide (**1**) was prepared by reaction of cysteamine with di-*tert*-butyl-1-(*tert*-buylthio)-1,2-hydrazinedicarboxylate as described previously [15]. After, dimethoxytritylthymidine (DMT-T, **2**) was prepared by standard protocols. Then, the 3’-OH of DMT-T (**2**) was protected with the transient trimethylsilyl group [18]. Activation of position 4 of 5’-DMT-3’-trimethylsilyl-T was done with a solution of phosphoryl *tris*(1,2,4-triazolide), previously prepared by mixing phosphoryl chloride and 1,2,4-triazole in the presence of a large excess of triethylamine [18,19,20]. The resulting triazolyl derivative was reacted with 2-aminoethyl-*tert*-butyl disulfide (**1**) yielding the desired 5’-DMT-2’-deoxy-*N*^4^-[2-(t-butyldisulfanyl)ethyl]-5-methylcytidine derivative (**3**). Compound **3** was reacted with 2-cyanoethoxy-*N*,*N*-diisopropylaminochlorophosphine yielding the desired phosphoramidite **4. **This phosphoramidite was incorporated into oligonucleotide sequence (5’-d (TTCCAXATTACCG)-3’ being X the position of the *N*^4^- [2-(t-butyldisulfanyl)ethyl]-5-methylcytidine derivative. The addition of the new phosphoramidite proceeded with 95-99% coupling yields, similar to standard phosphoramidites. After the assembly of the sequence, ammonia deprotection yielded the desired oligonucleotide together with a more polar side compound that had 40 mass units less than the desired oligonucleotide (see below) 

In order to determine the structure of the side compound a solid support functionalized with the protected 5’-DMT-2’-deoxy-*N*^4^-[2-(t-butyldisulfanyl)ethyl]-5-methylcytidine derivative was prepared. Compound **3** was then reacted with succinic anhydride to yield the 3’-hemisuccinate derivative that was reacted with amino-controlled pore glass (long chain amino alkyl-controlled pore glass, LCAA-CPG) yielding CPG solid support functionalized with 5’-DMT-2’-deoxy-*N*^4^-[2-(t-butyldisulfanyl)ethyl]-5-methylcytidine (**5**).

At this point we tested the stability of the disulfide bond of DMT-^Me^C(SS^t^Bu)-CPG (**5**) in the presence of the iodine solution (0.02 M iodine in water/ pyridine/ tetrahydrofuran) used during oligonucleotide synthesis. Alternatively the stability of the disulfide bond to a 10% solution of *tert*-butylhydroperoxide in acetonitrile was studied. For comparison purposes we also studied the stability of the disulfide bond of the 2’-deoxycytidine derivative DMT-C(SS^t^Bu)-CPG (**6**). This solid support was prepared as described [15].

**Scheme 2:**
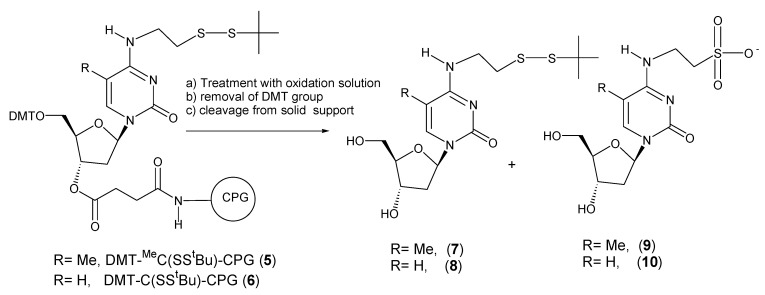
Products obtained after treatment of DMT-^Me^C(SS^t^Bu)-CPG (**5**) and DMT-C(SS^t^Bu)-CPG (**6**) with iodine or *tert*-butylhydroperoxide solutions. Reagents and conditions: **(a)** iodine or *tert*-butylhydroperoxide solution. **(b)** 3% trichloroacetic acid in dichloromethane. **(c) **concentrated ammonia, room temperature, 30 min.

Both DMT-^Me^C(SS^t^Bu)-CPG (**5**) and DMT-C(SS^t^Bu)-CPG (**6**) were treated with iodine solution for 5 min and 1 hr and *tert*-butylhydroperoxide for 3 h. During oligonucleotide synthesis, solid supports are exposed to iodine solution for approx. 1 min per cycle and to *tert*-butylhydroperoxide for 15 min per cycle. After the treatment with the oxidation solution, the DMT group was removed with trichloroacetic acid and the support treated with ammonia. The resulting nucleosides were analyzed by HPLC. Results are shown in Scheme 2 and Table 1.

**Table 1:** Products resulting from the treatment of DMT-^Me^C(SS^t^Bu)-CPG (**5**) and DMT-C(SS^t^Bu)-CPG (**6**) with iodine or *tert*-butylhydroperoxide solutions.

Solid Support	Treatment	^t^BuSS- protected / oxidized (-SO_3)_
**5**	Iodine solution, 5 min	95 : 5
**5**	Iodine solution, 1 h	3 : 97
**5**	*tert*-Butylhydroperoxide solution, 3 h	100 : 0
**6**	Iodine solution, 5 min	100 : 0
**6**	Iodine solution, 1 h	96 : 4
**6**	*tert*-Butylhydroperoxide solution, 3 h	100: 0

Treatment of solid support carrying 5’-DMT-2’-deoxy-*N*^4^-[2-(t-butyldisulfanyl)ethyl]-5-methylcytidine derivative (**5**) with iodine resulted on the formation of two nucleoside derivatives: the expected 2’-deoxy-*N*^4^-[2-(t-butyldisulfanyl)ethyl]-5-methylcytidine (**7**) (retention time 13.6 min, M = 388) and a new nucleoside derivative (retention time 6.2 min, M = 348). HPLC profiles are shown as supplementary material. The new compound was identified as the sulfonic acid derivative **9** resulting from the removal of the *t*-butylthio group, followed by oxidation of the resulting thiol group to the corresponding sulfonic acid. A 5-min iodine treatment (corresponding to five synthesis cycles) resulted on the formation of 5% of this side compound while 1 h treatment (60 synthesis cycles) resulted on the total conversion of the protected nucleoside to the oxidation product. The use of *tert*-butylhydroperoxide solution prevented the oxidation reaction (3 h treatment equivalent to 12 synthesis cycles). 

On the other hand treatment of solid support carrying 5’-DMT-2’-deoxy-*N*^4^-[2-(t-butyldisulfanyl)ethyl]cytidine derivative **6**, either with iodine or *tert*-butylhydroperoxide yielded the expected ^t^BuS protected nucleoside as the major compound (> 96%). It has been also described that ^t^BuS protected cysteine is stable to iodine and *tert*-butylhydroperoxide solutions [21,22]. We hypothesize that the electron donor properties of the methyl group at position 5 is responsible of a slightly higher electron density on the thiol group that may facilitate the oxidation of the sulfur with iodine with subsequent loss of the ^t^BuS group. Although some steric or other effects should be also present as the methyl group at C5 and the sulfur atom are separated by five bonds. 

Next we studied the stability of the ^t^BuS protected nucleosides on a dinucleotide sequence (DMT-C^Bz^p^Me^C(SS^t^Bu)-CPG (**11**) and DMT-C^Bz^pC(SS^t^Bu)-CPG (**12**)) to determine the effect a phosphate group in an environment similar to the one found inside of an oligonucleotide sequence. We used a similar protocol to the one described for the monomer except for the ammonia treatment that was performed at 50 ºC for 6 h (standard conditions for removal of nucleobase protecting groups).

**Scheme 3:**
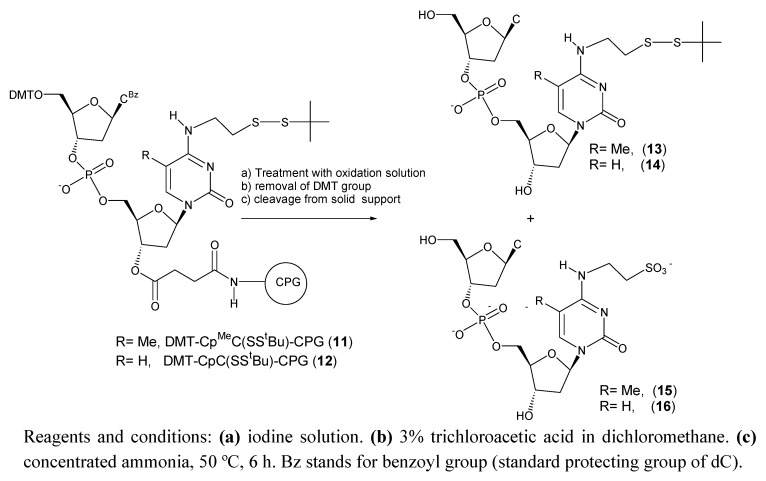
Products obtained after treatment of DMT-C^Bz^p^Me^C(SS^t^Bu)-CPG (**11**) and DMT-C^Bz^pC(SS^t^Bu)-CPG (**12**) with iodine solution.

Table 2 shows the results obtained in this study. The formation of the oxidation products were also observed but in a much lower efficiency. For example 1hr treatment of DMT-Cp^Me^C(SS^t^Bu)-CPG (**11**) with iodine solution yielded 19% of side compound (Table 2). The same treatment on DMT-^Me^C(SS^t^Bu)-CPG (**5**) gave 97% of side product (Table 1). A treatment of 3 h equivalent to 36 synthesis cycles generated a 38% of side compound (Table 2). As observed in the monomer the side reaction is less efficient with the cytosine derivative than the 5-methylcytosine derivative. A treatment of 3 h of DMT-CpC(SS^t^Bu)-CPG (**12**) with iodine solution generated a 12% of side compound instead of 38% that was observed for the support **11** (Table 2). In summary the presence of a nucleotide attached at the 5’ position slows the efficiency of the oxidation of ^t^BuS protected nucleoside to sulfonic acid. 

**Table 2:** Products resulting from the treatment of DMT-Cp^Me^C(SS^t^Bu)-CPG (**11**) and DMT-CpC(SS^t^Bu)-CPG (**12**) with iodine solution.

Solid Support	Treatment	^t^BuSS- protected / oxidized (-SO_3_)
**11**	Iodine solution, 5 min	96 : 4
**11**	Iodine solution, 1 h	81 : 19
**11**	Iodine solution, 3 h	62 : 38
**12**	Iodine solution, 5 min	100 : 0
**12**	Iodine solution, 1 h	93 : 7
**12**	Iodine solution, 3 h	88: 12

Next we studied the effect of the iodine or *tert*-butylhydroperoxide solutions in the purity of a short oligonucleotide containing one modified nucleoside in the middle of the sequence. Oligonucleotide sequence 5’-d(TTCCAXATTACCG)-3’ (being X the position of the *N*^4^- [2-(t-butyldisulfanyl)ethyl]-5-methylcytidine derivative) was prepared on 200 nmol scale using three different methods: a) LV200^®^ polystyrene support using iodine solution; b) LV200^®^ polystyrene support using *tert*-butylhydroperoxide solution and c) CPG support using iodine solution.

**Scheme 4:**
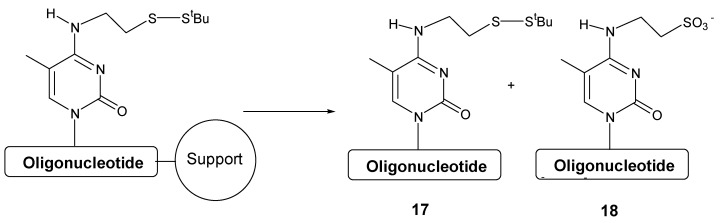
Potential products produced during the synthesis of oligonucleotides carrying *N*^4^- [2-(*t*-butyldisulfanyl)ethyl]-5-methylcytosine.

**Table 3:** Products resulting from the synthesis of 5’-d(TTCCAXATTACCG)-3’.

Solid Support	Oxidation solution	^t^BuSS- protected 17 / oxidized 18
polystyrene	Iodine	41 : 59
polystyrene	*tert*-Butylhydroperoxide	1 : 99
CPG	Iodine solution	6 : 94

As it can be seen in Table 3, the oxidation reaction is more pronounced on polystyrene support when iodine solution is used obtaining a 59% of the oligonucleotide carrying the sulfonic acid group **18**. When using polystyrene and *tert*-butylhydroperoxide solution as well as CPG and iodine solution only 1% and 6% of side compound **18** was observed. The formation of the side compound when using polystyrene support and iodine solution may be due to an increase of the local concentration of iodine by absorption of iodine on the polystyrene surface. 

**Figure 1:**
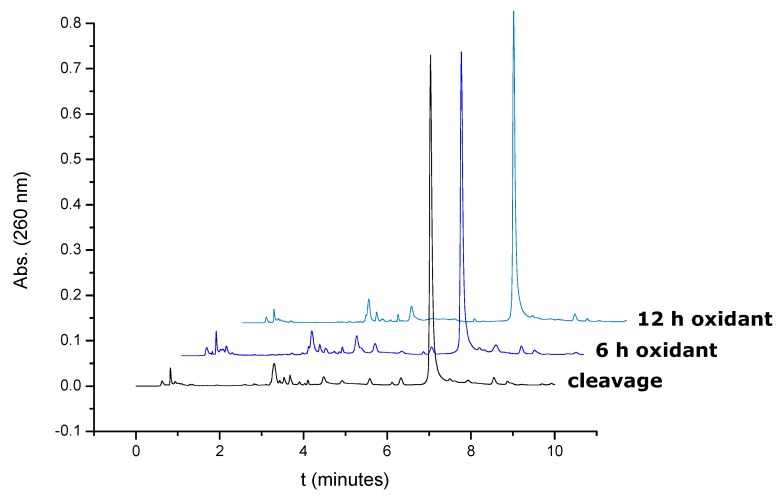
HPLC profiles of oligonucleotide 5’-d(TTCCAXATTACCG)-3’ after synthesis and after prolonged treatment of the support with *tert*-butylhydroperoxide solution for 6 and 12 h.

In order to analyze the stability of the disulfide bond in the presence of *tert*-butylhydroperoxide , the oligonucleotide attached to polystyrene and oxidized with *tert*-butylhydroperoxide was treated with the *tert*-butylhydroperoxide solution for 6 and 12 h more. The resulting crudes were analyzed by HPLC. Figure 1 shows that the disulfide bond is stable to *tert*-butylhydroperoxide solution at prolonged periods of time as no increase of the peaks around the elution of the oxidation product was observed. 

**Scheme 5:**
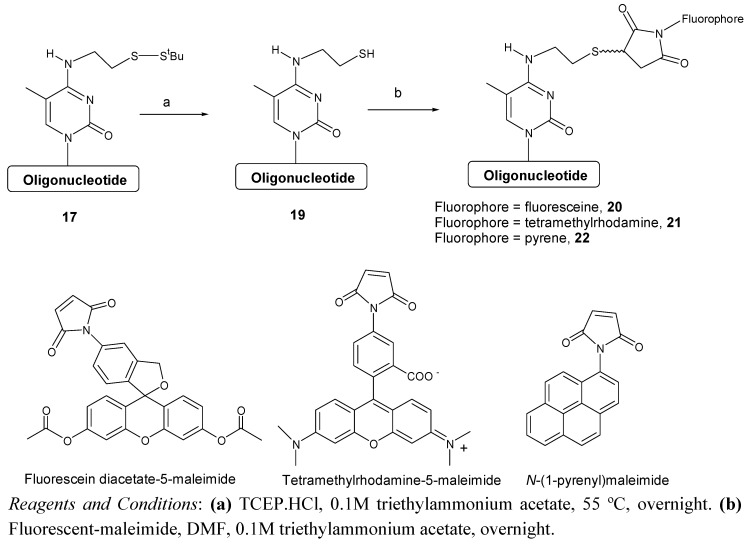
Synthesis of oligonucleotides carrying fluorescent molecules.

Finally, the use of the modified oligonucleotide for the preparation of oligonucleotides carrying fluorescent compounds was demonstrated. To this end oligonucleotide **17 **was treated with tris(2-carboxyethyl)phosphine to remove the ^t^BuS group and the resulting free thiol oligonucleotide **19** was reacted with three different fluorescent maleimides (Scheme 5). The desired fluorescent oligonucleotides were obtained as a mixture of isomers that were characterized by UV and mass spectrometry analysis. In the case of the fluorescein diacetate 5-maleimide we observed that the acetate groups are partially hydrolyzed in the reaction conditions yielding a complex HPLC profile (see supplementary material). A short treatment with a bicarbonate solution resulted on the total hydrolysis of the acetate groups obtaining a more simple HPLC profile. Coupling efficiency based on the integration of the HPLC peaks was between 80-84% (Table 4). 

**Table 4:** Oligonucleotide carrying fluorescent compounds prepared in this study.

Compound	Fluorophore	Yield (%)*	MS (expected)	MS (found)
**20**	Fluoresceine	82	4372.1	4378.6 / 4369.4^#^
**21**	Tetramethylrhodamine	80	4424.2	4422.6
**22**	Pyrene	84	4240.0	4240.5^#^

*Yield determined by the areas of the peaks in the HPLC chromatograms. ^#^Several isomers in HPLC.

## 3. Experimental

### 3.1. General

All reagents were purchased from Aldrich, Sigma or Fluka (Sigma-Aldrich Química S.A., Spain) and were used without further purification. Phosphoramidites and ancillary reagents used during oligonucleotide synthesis were from Applied Biosystems (PE Biosystems Hispania S.A., Spain). Flash column chromatography was carried out on silica gel SDS 0.063-0.2 mm/70-230 mesh. NMR spectra were recorded on a Varian Mercury 400 spectrometer operating at 400 MHz (^1^H) and 100 MHz (^13^C). Chemical shifts are reported in ppm relative to the singlet at *δ* = 7.24 ppm of CHCl_3_ for ^1^H-NMR and to the centre line of the triplet at *δ* = 77.0 ppm of CDCl_3_ for ^13^C-NMR. J values are given in Hz. ^31^P-NMR spectra were recorded on a Varian Inova 300 spectrometer. HPLC separations were performed using a Waters HPLC system. MALDI mass spectrometry was recorded on a Fisons VG Tofspec spectrometer and ESI mass spectra on a Fisons VG Platform II spectrometer. Oligonucleotide sequences were prepared using solid phase methodology. The syntheses of oligonucleotides were carried out on an Applied Biosystems Model 3400 DNA synthesizer. Details of the synthesis of 2-aminoethyl-tert-butyl disulfide (**1**) [15] and DMT-T (**2**) are described as supplementary material. HPLC profiles are shown as supplementary material.

### 3.2. Synthesis of 2’-deoxy-5’-O-(4,4’-dimethoxytriphenylmethyl)-N^4^-(tert-butyldithio-ethyl)-5-methyl-cytidine ***(3)***

DMT-T (**2**, 1.25 g, 2.30 mmol) was dried by evaporation of anhydrous ACN under reduced pressure, and the residue was dissolved in anhydrous DMF (30 mL) under argon. Hexamethyldisilazane (0.96 mL 4.6 mmol) was added with a syringe to the solution with exclusion of moisture. After 30 min of magnetic stirring at room temperature, the reaction was complete as judged by TLC (ethyl acetate, R_f _= 0.61). Then, the solution was evaporated under reduced pressure. The residue was dissolved in toluene (4 × 10 mL) and concentrated to dryness. 1,2,4-Triazole (1.67 g, 24.15 mmol) was dried by evaporation of anhydrous ACN under reduced pressure, and the residue was dissolved in anhydrous ACN (40 mL) under argon. Triethylamine (3.7 mL, 26.45 mmol) was added and the solution was cooled on ice. Then, phosphorus oxychloride (0.53 mL, 5.75 mmol) was added with a syringe to the solution with exclusion of moisture. After 30 min of magnetic stirring at T = 0 ºC, the protected nucleoside dissolved in anhydrous ACN (40 mL) was added dropwise to the solution under argon. The reaction mixture was stirred at room temperature. After 6 hours, the reaction was complete as judged by TLC (ethyl acetate, R_f _= 0.39). The solvent was removed under reduced pressure and the residue dissolved in DCM (50 mL). The solution was washed with saturated aqueous NaCl (50 mL). After drying the organic phase with Na_2_SO_4_, the solvent was evaporated under reduced pressure. The residue was dissolved in dry DMF (20 mL) under argon. Compound **1** (0.57 g, 3.45 mmol) and DBU (0.52 mL, 3.45 mmol) were added to the solution. The reaction mixture was stirred overnight at room temperature. DBU was added (0.34 mL, 2.30 mmol) and the reaction mixture was stirred at room temperature. After 6 hours, the solvent was removed under reduced pressure and the residue was dissolved in ethyl acetate (100 mL). The solution was washed with saturated aqueous sodium chloride (100 mL). After drying the organic phase with sodium sulphate, the solvent was evaporated under reduced pressure. The residue was dissolved in a small amount of ethyl acetate and purified by chromatography on silica gel. The column was packed with silica gel using a 1% triethylamine solution in ethyl acetate. The product was eluted with a gradient of methanol from 0 to 5% in ethyl acetate. The pure compound was obtained as pale yellow foam (0.85 g, 53%). TLC (3% methanol in ethyl acetate) R_f _= 0.25. ^1^H-NMR, δ_H_ (CDCl_3_): 7.61 (s, 1H), 7.45-6.70 (m, 13H), 6.38 (t, 1H, *J *= 6.6 Hz), 5.28 (t, 1H, *J *= 5.6 Hz), 4.50-4.47 (m, 1H), 4.04-4.01 (m, 1H), 3.79 (t, 2H, *J* = 6.0 Hz), 3.72 (s, 6H), 3.40 (dd, 1H, *J *= 10.4 and 3.2 Hz), 3.28 (dd, 1H, *J* = 10.6 and 3.0 Hz), 2.84 (t, 2H, *J* = 6.0 Hz), 2.55-2.50 (m, 1H), 2.20-2.13 (m, 1H), 1.43 (s, 3H), 1.26 (s, 9H). ^13^C-NMR, δ_C_ (CDCl_3_): 163.27 (C), 158.82 (CH), 156.54 (C), 144.76 (C), 137.77 (CH), 135.85 (C), 130.33 (CH), 128.39 (CH), 128.15 (CH), 127.21 (C), 113.45 (CH), 102.29 (C), 86.91 (C), 86.17 (CH), 86.16 (CH), 72.42 (CH), 63.83 (CH_2_), 55.48 (CH_3_), 48.53 (C), 42.30 (CH_2_), 39.87 (CH_2_), 39.27 (CH_2_), 30.11 (CH_3_), 12.65 (CH_3_); ESI-MS m/z (+ve mode [M+H]^+^) calc. for C_37_H_45_N_3_O_6_S_2_ 691.90, found 692.28.

### 3.3. Synthesis of 2’-deoxy-5’-O-(4,4’-dimethoxytriphenylmethyl)-N^4^-(2-aminoethyldi-thioethyl)-5-methylcytidine-3-O-(2-cyanoethyl-N,N’-diisopropylphosphoramidite) ***(5)***

Compound **3** (200 mg, 0.22 mmol) was dried by evaporation of anhydrous ACN *in vacuo*, and the residue was dissolved in anhydrous DCM (10 mL) under argon. *N*,*N*’-diisopropylethylamine (154 µL, 0.88 mmol) was added with exclusion of moisture. The solution was cooled on ice and 2-cyanoethoxy-*N*,*N*’-diisopropylaminochlorophosphine (73 µL, 0.33 mmol) was added dropwise with a syringe. Afterward, the solution was stirred at room temperature for 1 h. After this time, TLC (1% cyclohexane in ethyl acetate) showed the presence of the starting compound. As a consequence more 2-cyanoethoxy-*N*,*N*-diisopropylaminochlorophosphine (26 μL, 0.11 mmol) once the mixture was cooled on ice. After the addition the reaction mixture was allowed to stir at room temperature for 1 h. The solvent was removed in vacuo and the residue was dissolved in dichloromethane (20 mL). The solution was washed with 5% aqueous sodium hydrogen carbonate (20 mL) and with saturated aqueous sodium chloride (20 mL). After drying the organic phase with sodium sulphate, the solvent was evaporated under reduced pressure. The residue was dissolved in a small amount of ethyl acetate/cyclohexane 2:1 and purified by chromatography on silica gel. The column was packed with silica gel using a 5% triethylamine solution in ethyl acetate/cyclohexane 2:1. The product was eluted with ethyl acetate/cyclohexane 2:1. The pure compound was obtained as pale yellow foam (220 mg, 86%). TLC (1% cyclohexane in ethyl acetate) R_f _= 0.39 and 0.26. ^1^H-NMR, δ_H_ (CDCl_3_): Most of signals are duplicated due to the presence of diastereoisomers 7.72 and 7.65 (2s, 1H), 7.43-6.81 (m, 13H), 6.47 and 6.43 (2t, 1H, *J *= 6.4 and 6.4 Hz respectively), 4.66-4.57 (m, 1H), 4.23-4.01 (m, 1H), 3.87-3.91 (m, 2H), 3.80 and 3.79 (2s, 6H), 3.61-3.46 (m, 4H), 2.93 (t, 2H, *J *= 5.8 Hz), 2.76 (td, 2H, *J *= 6.2 and 2.0 Hz), 2.66-2.56 (m, 1H), 2.61 and 2.38 (2t, 1H, *J *= 6.4 and 6.4 Hz respectively), 2.30-2.20 (m, 1H), 1.46 and 1.43 (2s, 3H), 1.34 (s, 9H), 1.29-1.14 (m, 12H). ^13^C-NMR, δ_C_ (CDCl_3_): Most of signals are duplicated due to the presence of diastereoisomers 163.24 and 163.22 (C), 158.89 and 158.87 (CH), 156.37 and 156.33 (C), 144. 63 (C), 137.65 and 137.60 (CH), 135.75 and 135.73 (C), 130.46 and 130.37 (CH), 128.56 and 128.46 (CH), 128.13 (CH), 127.29 and 127.25 (C), 117.81 (C), 113.49 (C), 113.42 (CH), 102.18 and 102.06 (C), 86.90 and 86.88 (C), 85.79 (CH), 85.40 and 85.28 (2d, *J *= 4.7 and 5.8 Hz), (CH), 73.78 and 72.76 (2d, *J* = 8.2 and 7.2 Hz) (CH), 63.34 and 62.84 (CH_2_), 58.58 and 58.37 (2d, *J* = 10.9 and 11.3 Hz) (CH_2_), 55.49 and 55.46 (CH_3_), 48.52 (C), 45.57 and 45.51 (CH_2_), 43.49 and 43.37 (2d, *J *= 9.2 and 9.7 Hz) (CH), 43.41 and 43.32 (CH), 39.81 (CH_2_), 39.32 (CH_2_), 30.11 (CH_3_), 24.76 and 24.75 (2d, *J* = 8.2 Hz and 7.2 Hz) (CH_3_), 23.17 and 23.14 (2d, *J* = 8.2 and 8.7 Hz) (CH_3_), 20.35 and 20.33 (2d, *J *= 7.1 and 6.9 Hz) (CH_2_), 12.57 and 12.55 (CH_3_). ^31^P NMR, δ_P_ (CDCl_3_, 81 MHz): 150.03 and 149.61, two diastereoisomers. ESI-MS m/z (+ve mode) [M+H]^+ ^= 892.40, (M = 892.12 g/mol calculated for C_46_H_62_N_5_O_7_PS_2_).

### 3.4. Preparation of the solid support functionalized with 2’-deoxy-5’-O-(4,4’-dimethoxytriphenyl-methyl)-N^4^-(2-aminoethyldithioethyl)-5-methylcytidine (DMT-^Me^C(SS^t^Bu)-succinyl-CPG) ***(5)***

5’-DMT-*N*^4^-(2-aminoethyldithioethyl)-5-methylcytidine was incorporated on a long-chain alkylamine-controlled pore glass support (LCAA-CPG), following the standard methodology via monosuccinate derivative **4**. Compound **3** (50 mg, 0.07mmol) was dried by evaporation of anhydrous ACN under reduced pressure, and the residue was dissolved in anhydrous pyridine (5 mL) under argon. Succinic anhydride (18 mg, 0.18 mmol) and 4-dimethylaminopyridine (DMAP) (5 mg, 0.04 mmol) were added to the solution. After 4 hours of magnetic stirring at room temperature, the reaction was complete as judged by TLC (5% methanol in ethyl acetate) R_f _= 0.11. The solvent was removed under reduced pressure and the residue was dissolved in DCM (20 mL). The solution was washed with saturated aqueous sodium chloride (15 mL). After drying the organic phase with sodium sulphate, the solvent was evaporated under reduced pressure. The monosuccinate derivative **4**, which was used in the next step without further purification, was obtained as a pale yellow foam. 250 mg of commercial LCAA-CPG (CPG New Jersey, 73 µmol amino/g) were placed into a polypropylene syringe fitted with a polypropylene disc and washed sequentially with DMF, methanol, THF, DCM, and ACN. Then, a solution of 5’-DMT-N^4^-(2-aminoethyldithioethyl)-5-methylcytidine-3’-mono-succinate (**4**, 22 mg, 27 µmol), hydroxybenzotriazole (HOBt) (4 mg, 27 µmol) and diisopropyl-carbodiimide (DIP) (23 µL, 146 µmol) in 700 µL of DMF was prepared. The solution was added to the resin and left to react for 2 h. The resin was washed with DMF, methanol, DCM and ACN. After washings, the resin was treated with 500 µL of Ac_2_O/DMF 1:1 to cap free amino groups. The incorporation of nucleoside was determined by DMT quantification (34 µmol/g).

### 3.5. Study of the stability of DMT-^Me^C(SS^t^Bu)-CPG under oxidative conditions and comparison with DMT-C(SS^t^Bu)-CPG

The solid support functionalized with the cytidine derivative, 5’-DMT-*N*^4^-(2-aminoethyldithioethyl)cytidine (DMT-C(SS^t^Bu)-CPG) (**6**) was synthesized as described in [15]. Five mg of each solid support (DMT-C(SS^t^Bu)-CPG) (**6**) and (DMT-^Me^C(SS^t^Bu)-CPG) (**5**) were treated with oxidation solutions (iodine or *t*-butylhydroperoxide). Iodine solution was the same solution used in synthesis of oligonucleotides (0.02 M iodine in water/ pyridine/ tetrahydrofuran). The 10% *t*-butylhydroperoxide solution was prepared freshly by mixing 86.4 mL of acetonitrile and 14.3 mL of commercially available 70% *t*-butylhydroperoxide solution in water. At different time intervals the solution were filtered and the resulting solid support washed with ACN. The resulting supports were treated with a solution of trichloroacetic acid 3% in DCM for 5 min to remove the DMT group and with aqueous concentrated ammonia for 30 min to cleave the product from the resin. The mixture was filtered and the ammonia solution was concentrated to dryness. The resulting products were analyzed by HPLC (Column: X-Bridge ^TM^OST C_18_ (2.5 μm; 4.6 × 50 mm); solvent A: 100 mM triethylammonium acetate (pH = 7) and solvent B: 70% ACN in 100 mM triethylammonium acetate (pH = 7). Flow rate: 1 mL/min. Conditions: 2.5 min 100% A, then 5.5 min linear gradient from 0-12.5% B, then 12 min linear gradient from 12-100% B, and mass spectrometry. HPLC profiles are shown as supplementary material. Compound **7**: t_R _= 13.6 min, ESI-MS m/z (negative mode [M-H]^-^) calc for C_16_H_27_N_3_O_4_S_2_ 389.53, found 388.14. Compound **8**: t_R _= 13.2 min, ESI-MS m/z (negative mode [M-H]^-^) calc for C_15_H_25_N_3_O_4_S_2_ 375.51, found 373.17. Compound **9**: t_R _= 6.2 min, ESI-MS m/z (negative mode [M-H]^-^) calc for C_12_H_19_N_3_O_7_S 349.35, found 348.08. Compound **10**: t_R _= 4.7 min, ESI-MS m/z (negative mode [M-H]^-^) calc for C_11_H_17_N_3_O_7_S 335.33, found 334.08.

### 3.6. Analysis of the stability of DMT-Cp(^Me^C(SS^t^Bu))-CPG and DMT-Cp(C(SS^t^Bu))-CPG solid supports to oxidative conditions

DMT-dC^Bz^ phosphoramidite was incorporated in the solid supports **5** and **6** previously synthesized in a DNA synthesizer (Applied Biosystems 3400) on 1 μmol scale using commercially available chemicals. The synthesis was carried out using the standard phosphite triester methodology. For the oxidation step was used a solution of *tert*-butyl hydroperoxide 10% in ACN instead of the commercially available solution of iodine 0.02 M. In both cases the synthesis was carried out without the removal of the 5’-DMT group.

The analysis of the stability of the thiolated nucleosides under oxidation conditions were studied as described for supports **5** and **6**. Five mg of each solid support were treated with iodine solution. At different time intervals the solution were filtered and the resulting solid support washed with acetonitrile. The resulting supports were treated with a solution of trichloroacetic acid 3% in DCM for 5 min to remove the DMT group and with aqueous concentrated ammonia at 55 ºC for 6 h to cleave the products from the support and to remove the benzoyl group. The mixtures were filtered and the ammonia solutions were concentrated to dryness. The resulting products were characterized by HPLC (Column: X-Bridge ^TM^OST C_18_ (2.5 μm, 4.6 × 50 mm); solvent A: 100 mM triethylammonium acetate (pH = 7) and solvent B: 70% ACN in 100 mM triethylammonium acetate (pH = 7). Flow rate: 1 mL/min. Conditions: 2.5 min 100% A, then 5.5 min linear gradient from 0-12.5% B, then 12 min linear gradient from 12-100% B, and mass spectrometry. HPLC profiles are shown as supplementary material. Compound **13**: t_R _= 12.4 min, ESI-MS m/z (negative mode [M-H]^-^) calc for C_25_H_39_N_6_O_10_PS_2_ 678.71, found 677.17. Compound **14**: t_R _= 12.2 min, ESI-MS m/z (negative mode [M-H]^-^) calc for C_24_H_37_N_6_O_10_PS_2_ 664.68, found 663.18. Compound **15**: t_R _= 7.6 min, ESI-MS m/z (negative mode [M-H]^-^) calc for C_21_H_31_N_6_O_13_PS 638.53, found 637.13. Compound **16**: t_R _= 7.0 min, ESI-MS m/z (negative mode [M-H]^-^) calc for C_20_H_29_N_6_O_13_PS 624.50, found 623.13.

### 3.7. Synthesis of oligonucleotides containing a residue of N^4^-mercaptoethyl-5-methylcytosine

The oligonucleotide with the sequence ^5’^TTCCAXATTACCG^3’^, where X stands for the modified nucleoside, was synthesized on 0.2 µmol scale employing three methodologies:

a) LV200^®^ polystyrene, iodine solution, 1 min.
b) CPG; iodine solution, 1 min.
c) LV200^®^ polystyrene, 10 % of *tert*-butylhydroperoxide in ACN, 15 min.

The protecting group for dC and dA was the benzoyl (Bz) group. The isobutyryl (^i^bu) group was used for protection of dG. In all cases, at the end of the synthesis the DMT group was removed. The coupling efficiency for the modified phosphoramidite was between 95-99% (as measured by the absorbance of the DMT group). After the assembly of the sequence the solid supports were treated with concentrated aqueous ammonia for 6 h at 55 ºC. Under these conditions, all base and phosphate groups were also removed. The mixtures were filtered and the ammonia solutions were concentrated to dryness. The resulting crudes were analyzed by HPLC (Column: X-Bridge ^TM^OST C_18_ (2.5 μm; 4.6 × 50 mm); solvent A: 5% ACN in 100 mM triethylammonium acetate (pH = 7) and solvent B: 70% ACN in 100 mM triethylammonium acetate (pH = 7). Flow rate: 1 mL/min. Conditions: 10 min linear gradient from 0-30%B. The different peaks observed in the HPLC profiles were analyzed by mass spectrometry. HPLC profiles are shown as supplementary material. Compound **17**: t_R _= 7.5 min, MALDI-TOF m/z (negative mode [M-H]^-^) calc for C_132_H_175_N_43_O_77_P_12_S_2_ 4031.90, found 4032.52. Compound **18**: t_R _= 4.9 min, ESI-MS m/z (negative mode [M-H]^-^) calc for C_128_H_166_N_6_O_80_P_12_S 3991.80, found 3996.15.

### 3.8. Synthesis of oligonucleotide-fluorophore conjugates

The oligonucleotide obtained before was used to perform the reactions with fluorescein diacetate 5-maleimide, tetramethylrhodamine-5-maleimide and *N*-(1-pyrenyl)maleimide For the preparation of each conjugate, 1.4 OD_260_ units of oligonucleotide **17** were used. To cleave the disulfide bond, compound **17** was dissolved in 0.3 mL of 0.1 M triethylammonium acetate solution (pH = 7). Afterward, 34 μL of a 0.1 M tris(2-carboxyethyl)phosphine hydrochloride (TCEP. HCl) solution were added to the solution and allow to react at 55 ºC overnight. Under these conditions, the *tert*-butylthiol group was completely removed. The resulting product was purified with Sephadex G-25 (NAP-5 column). The oligonucleotide **19** was eluted with 1 mL of sterile water. The solution was analyzed by HPLC (Column: X-Bridge ^TM^OST C_18_ (2.5 μm, 4.6 × 50 mm); solvent A: 5% ACN in 100 mM triethylammonium acetate (pH = 7) and solvent B: 70% ACN in 100 mM triethylammonium acetate (pH = 7). Flow rate: 1 mL/min. Conditions: 10 min linear gradient from 5-40% B. Under these conditions the protected oligonucleotide eluted at t_R _= 4.9 min and the deprotected oligonucleotide at t_R _= 3.0 min. The solution was concentrated to 500 μL and 50 μL of a 1M triethylammonium acetate solution were added. Then, 100 μL of a solution 0.56 mM fluorescein diacetate 5-maleimide, tetramethylrhodamine-5-maleimide or *N*-(1-pyrenyl)maleimide in DMF were added and allowed to react at room temperature overnight. The crude oligonucleotide was concentrated to dryness and the excess of reagents and salts were removed by using a NAP-5 column. The oligonucleotide was eluted with 1 mL of sterile water. All crudes were analyzed by HPLC using the conditions described above. HPLC profiles are shown as supplementary material.

#### 3.8.1. Conjugation with fluorescein diacetate 5-maleimide

The HPLC analysis showed the presence of four products related with the oligonucleotide-fluoresecein conjugate with t_R _= 6.6 min, (λ_max _= 262 and 451 nm), t_R _= 4.1 min, (λ_max _= 262 and 493 nm), t_R _= 3.6 min, (λ_max _= 262 and 490 nm) and t_R _= 3.4 min, (λ_max _= 262 and 486 nm). The analysis of all four bands by EM (MALDI-TOF) indicated that the high t_R_ band corresponded to the monodeacetylated form of the oligonucleotide-fluorescein conjugate (**20d**). The band collected at t_R _= 4.1 min corresponded to the diacetylated form of the conjugate (**20c**). The other two bands (**20a** and **20b**) shared the same mass found in **20c**. To the resulting crude of synthesis were added 100 μL of a solution of 2 M sodium bicarbonate and left to react for 1 h at 55 ºC. The crude oligonucleotide was concentrated to dryness and the excess of salts were removed by using a NAP-5 column. The oligonucleotide was eluted with 1 mL of sterile water. The HPLC analysis of the crude revealed the presence of only two products related with the oligonucleotide-fluorescein conjugate, **20a** and **20b**. The coupling efficiency was determined from the HPLC profiles (~82%). Compound **20a**: t_R _= 3.4 min, MALDI-TOF m/z (negative mode [M-H]^-^) calc for C_152_H_180_N_44_O_84_P_12_S 4372.08, found 4378.56. Compound **20b**: t_R _= 3.6 min, MALDI-TOF m/z (negative mode [M-H]^-^) calc for C_152_H_180_N_44_O_84_P_12_S 4372.08, found 4369.44. Compound **20c**: t_R _= 4.1 min, ESI-MS m/z (negative mode [M-H]^-^) calc for C_152_H_180_N_44_O_84_P_12_S 4372.08, found 4370.02. Compound **20d**: t_R _= 6.6 min, ESI-MS m/z (negative mode [M-H]^-^) calc for C_154_H_192_N_44_O_85_P_12_S 4414.12, found 4413.63.

#### 3.8.2. Conjugation with tetramethylrhodamine-5-maleimide

The HPLC analysis of the crude revealed the presence of four bands between t_R _= 7.6 min and t_R _= 8.3 min. Only the band with t_R _= 7.9 min showed an UV spectra consistent with an oligonucleotide (*λ*_max _= 262 nm) with rhodamine (λ_max _= 554 nm). The other three peaks showed *λ*_max_ around 556 nm without the maxima at 260 nm, indicating that they are tetramethylrhodamine-5-maleimide derivatives that could not be removed with a NAP-5 column. This is due to the positive charge of tetramethylrhodamine that interacts with the negative charge of the phosphate bonds. The conjugate was characterized as well by EM (MALDI-TOF). The coupling efficiency was determined from the HPLC profile (~80%). Compound **21**: t_R _= 7.9 min, ESI-MS m/z (negative mode [M-H]^-^) calc for C_156_H_189_N_46_O_82_P_12_S 4424.20, found 4422.58.

#### 3.8.3. Conjugation with *N*-(1-pyrenyl)maleimide

The crude was analyzed by HPLC showing at least four bands (t_R _= 6.9, 7.3, 7.7 and 7.8 min) sharing the same UV spectra (λ_max _= 263 and 341 nm) and the same mass by mass spectrometry. All four bands corresponded to the oligonucleotide-pyrene conjugate (**22**), being different isomers. The coupling efficiency was determined from the HPLC profile (~84%). Compound **22**: t_R _= 6.9, 7.3, 7.7 and 7.8 min, ESI-MS m/z (negative mode [M-H]^-^) calc for C_148_H_177_N_44_O_79_P_12_S 4240.01, found 4240.52.

## 4. Conclusions

The excellent molecular recognition properties of synthetic oligonucleotides are being exploited for the rational construction of 2-D DNA lattices with well defined structures [23,24]. A step ahead is to incorporate chemical groups at specific sites of 2-D DNA lattices to direct the deposition of specific molecules or nanomaterials [25] or to direct deposition to surfaces [17]. In this article we have described the synthesis of the 2’-deoxy-*N*^4^-[2-(t-butyldisulfanyl)ethyl]-5-methylcytidine phosphoramidite building block. This compound has been developed for the specific introduction of thiol groups at the nucleobase. During the use of the new building block a side reaction was observed. This side reaction has not been observed previously yielding a non reactive sulfonic acid. The side reaction is mostly observed in the 5-methylcytosine derivative and using iodine solution for the oxidation of phosphites on polystyrene supports. Replacement of iodine solution with a solution of *t*-butylhydroperoxide eliminates this side reaction. Several examples of the use of oligonucleotides containing *N*^4^-mercaptoethyl-5-methylcytosine for the preparation of fluorescent DNA conjugates are described.

## Supplementary Materials

Supplementary materials can be seen at http://www.mdpi.com/1420-3049/15/8/5692/s1.
