# Supplementary material for: Solid-Phase Synthesis of Oligodeoxynucleotides Containing N4-[2-(t-butyldisulfanyl)ethyl]-5-methylcytosine Moieties
Source: Molecules. 2010 Aug 18;15(8):5692–707. doi: 10.3390/molecules15085692 (PMC6257692; doi:10.3390/molecules15085692)
Supplement: Supplementary file 1 [file molecules-15-05692-s001.docx]

Supplementary material

Solid-Phase Synthesis of Oligodeoxynucleotides Carrying *N*^4^-[2-(*t*-butyldisulfanyl)ethyl]-5-methylcytosine

Sónia Pérez-Rentero, Alejandra V. Garibotti and Ramón Eritja*

Institute for Research in Biomedicine (IRB Barcelona), CIBER-BBN Networking Centre on Bioengineering, Biomaterials and Nanomedicine, Institute for Advanced Chemistry of Catalonia (IQAC) CSIC, Baldiri Reixac 10, E-08028 Barcelona, Spain.

CONTENTS

Synthesis of 2-aminoethyl-*tert*-butyl disulfide (**1**)

Synthesis of DMT-T (**2**)

Figure S1. HPLC profiles of A) ^Me^dC(SS^t^Bu), **7** and B) dC(SS^t^Bu), **8**

Figure S2. HPLC profiles of crudes A) DMT-^Me^C(SS^t^Bu)-CPG after treatment with an iodine solution (5 min and 1 h) and after treatment with a *tert*-buylhydroperoxide solution (3 h) and B) DMT-C(SS^t^Bu)-CPG after treatment with an iodine solution (5 min and 1 h) and after treatment with a *tert*-buylhydroperoxide solution (3 h).

Figure S3. HPLC profiles of A) dinucleoside **13** and B) dinucleoside **14**.

Figure S4. HPLC profiles of crudes A) DMT-Cp^Me^C(SS^t^Bu)-CPG after treatment with an iodine solution (5 min, 1 h and 3 h) and B) DMT-CpC(SS^t^Bu)-CPG after treatment with an iodine solution (5 min, 1 h and 3 h).

Figure S5. HPLC profiles of crude oligonucleotide **17**, A) solid support: polystyrene, oxidant: iodine, B) solid support: CPG, oxidant: iodine, and C) solid support: polystyrene, oxidant: *tert*-butylhydroperoxide.

Figure S6. HPLC profiles of A) protected oligonucleotide **17** and B) deprotected oligonucleotide **19**.

Figure S7. HPLC profiles of crude A1) oligonucleotide-fluorescein conjugate, A2) oligonucleotide-fluorescein conjugate after treatment with a bicarbonate solution, B) oligonucleotide-rhodamine conjugate and C) oligonucleotide-pyrene conjugate.

*Synthesis of 2-aminoethyl-tert-butyl disulfide (****1****).*

Di-*tert*-butyl-1-(*tert*-butylthio)-1,2-hydrazinedicarboxylate (5 g 15.6 mmol) and triethylamine (1.1 mL 7.8 mmol) were dissolved in anhydrous dimethylformamide (DMF) (30 mL) under argon. A solution of cysteamine hydrochloride (0.88 g 7.8 mmol) in dry DMF (30 mL) was prepared and added dropwise to the stirred solution. After stirring the reaction mixture at room temperature overnight, a white solid was precipitating. Dry DMF (20 mL) was added to obtain a nearly clear solution. Triethylamine was added until a white solid was precipitating again and the reaction mixture was stirred at room temperature. After 8 hours the solution was filtered and evaporated under reduced pressure. The residue was dissolved five times in diethyl ether (10 mL), stirred for several minutes and filtered. The desired product was obtained a white solid (0.93 g, 73%). TLC (1% methanol in dichloromethane) R_f_=0.20; ^1^H NMR, δ_H_ (CDCl_3_, 400 MHz): 8.44 (s, 3H), 3.37-3.33 (m, 2H), 3.06 (t, J=7 Hz, 2H), 1.35 (s, 9H); ^13^C NMR, δ_C_ (CDCl_3_, 100 MHz): 48.55 (C), 38.96 (CH_2_), 35.98 (CH_2_), 30.14 (CH_3_); ESI-MS m/z (positive mode) [M+H]^+^=166.07, (M=165.32 g/mol calculated for C_6_H_15_NS_2_).

*Synthesis of 5’-O-(4,4’-dimethoxytriphenylmethyl)thymidine (****2****)*

Thymidine (2.0 g. 8.3 mmol) was dried by evaporation of anhydrous acetonitrile (ACN) under reduced pressure and the solid was dissolved in anhydrous pyridine (40 mL) under argon. 4,4’-Dimethoxytriphenylmethyl chloride (2.8 g 9.1 mmol) was added with exclusion of moisture. The reaction mixture was stirred overnight at room temperature. Afterward the reaction was complete as judged by TLC and quenched with methanol (0.5 mL). The solvent was removed in vacuo and the residue was dissolved in dichloromethane (150 mL). The solution was washed with 5% aqueous sodium hydrogen carbonate (80 mL) and with saturated aqueous sodium chloride (80 mL). After drying the organic phase with sodium sulphate, the solvent was evaporated under reduced pressure. The residue was dissolved in a small amount of dichloromethane and purified by chromatography on silica gel. The column was packed with silica gel using a 1% triethylamine solution in dichloromethane. The product was eluted with a gradient of methanol from 0 to 2% in dichloromethane. The pure compound was obtained as white foam (3.8 g 84%). TLC (2% methanol in dichloromethane) R_f_=0.40.

^1^H NMR, δ_H_ (CDCl_3_, 400 MHz): 7.78 (d, 1H), 7.41-6.82 (m, 13H), 6.31 (t, 1H), 5.40 (d, 1H), 4.57-4.53 (m, 1H), 4.03-4.01 (m, 1H), 3.79 (s, 6H), 3.49-3.41 (m, 2H), 2.47-2.41 (m, 1H), 2.29-2.22 (m, 1H); ^13^C NMR, δ_C_ (CDCl_3_, 100 MHz): 164.13 (C), 158.93 (CH), 150.78 (C), 144.55 (C), 135.94 (CH), 135.64 (C), 130.30 (CH), 128.35 (CH), 128.21 (CH), 127.35 (C), 113.51 (CH), 111.51 (C), 87.15 (C), 86.49 (CH), 85.00 (CH), 72.73 (CH), 63.86 (CH_2_), 55.48 (CH_3_), 41.19 (CH_2_), 12.05 (CH_3_); ESI-MS m/z (positive mode) [M+Na]^+^=567.22, (M=544.59 g/mol calculated for C_31_H_32_N_2_O_7_).

Figure S1. HPLC profiles of A) ^Me^dC(SS^t^Bu), **7** and B) dC(SS^t^Bu), **8**

Figure S2. HPLC profiles of crudes A) DMT-^Me^C(SS^t^Bu)-CPG after treatment with an iodine solution (5 min and 1 h) and after treatment with a *tert*-buylhydroperoxide solution (3 h) and B) DMT-C(SS^t^Bu)-CPG after treatment with an iodine solution (5 min and 1 h) and after treatment with a *tert*-buylhydroperoxide solution (3 h). **+** ^t^BuS protected nucleoside **7** or **8**. *oxidized nucleoside possessing a sulfonic acid group **9** or **10**.

Figure S3. HPLC profiles of A) dinucleoside **13** and B) dinucleoside **14**.
Figure S4. HPLC profiles of crudes A) DMT-Cp^Me^C(SS^t^Bu)-CPG after treatment with an iodine solution (5 min, 1 h and 3 h) and B) DMT-CpC(SS^t^Bu)-CPG after treatment with an iodine solution (5 min, 1 h and 3 h). **+** ^t^BuS protected dimer **13** or **14**. *oxidized dimer possessing a sulfonic acid group **15** or **16**.

Figure S5. HPLC profiles of crude oligonucleotide 18, A) solid support:polystyrene, oxidant: iodine, B) solid support: CPG, oxidant: iodine, and C) solid support: polystyrene, oxidant: *tert*-butylhydroperoxide. **+** ^t^BuS protected oligonucleotide **17**. *oxidized oligonucleotide possessing a sulfonic acid group **18** ♦ truncated sequence 5’ATTACCG 3’ (MALDI-TOF, found 2084.61 expected 2080.40).

Figure S6. HPLC profiles of A) protected oligonucleotide **17** and B) deprotected oligonucleotide **19**.

Figure S7. HPLC profiles of crude A1) oligonucleotide-fluorescein conjugate, A2) oligonucleotide-fluorescein conjugate after treatment with a bicarbonate solution, B) oligonucleotide-rhodamine conjugate and C) oligonucleotide-pyrene conjugate.
